# Contribution of the Epstein-Barr virus to the oncogenesis of mature T-cell lymphoproliferative neoplasms

**DOI:** 10.3389/fonc.2023.1240359

**Published:** 2023-09-14

**Authors:** Mario Henrique M. Barros, Paula Daniela S. Alves

**Affiliations:** ^1^ Institute of Pathology, Klinikum Chemnitz, Chemnitz, Germany; ^2^ Oncovirology Laboratory, Bone Marrow Transplantation Center, Instituto Nacional de Câncer (INCA), Rio de Janeiro, RJ, Brazil

**Keywords:** Epstein-Barr virus, T-cell lymphoma, EBV-positive nodal T-and NK-cell lymphoma, extranodal NK/T-cell lymphoma, infectious mononucleosis, LMP1, HLA

## Abstract

EBV is a lymphotropic virus, member of the *Herpesviridae* family that asymptomatically infects more than 90% of the human population, establishing a latent infection in memory B cells. EBV exhibits complex survival and persistence dynamics, replicating its genome through the proliferation of infected B cells or production of the lytic virions. Many studies have documented the infection of T/NK cells by EBV in healthy individuals during and after primary infection. This feature has been confirmed in humanized mouse models. Together these results have challenged the hypothesis that the infection of T/NK cells *per se* by EBV could be a triggering event for lymphomagenesis. Extranodal NK/T-cell lymphoma (ENKTCL) and Epstein-Barr virus (EBV)-positive nodal T- and NK-cell lymphoma (NKTCL) are two EBV-associated lymphomas of T/NK cells. These two lymphomas display different clinical, histological and molecular features. However, they share two intriguing characteristics: the association with EBV and a geographical prevalence in East Asia and Latin America. In this review we will discuss the genetic characteristics of EBV in order to understand the possible role of this virus in the oncogenesis of ENKTCL and NKTCL. In addition, the main immunohistological, molecular, cytogenetic and epigenetic differences between ENKTCL and NKTCL will be discussed, as well as EBV differences in latency patterns and other viral molecular characteristics.

## Introduction

EBV is a lymphotropic virus, member of the *Herpesviridae* family that asymptomatically infects more than 90% of the human population (1). EBV enters the organism mainly via the oropharyngeal epithelium and infects circulating B cells to establish itself in a state of latency in the memory B cells. Its primoinfection occurs at variable ages, depending on the socioeconomic conditions of the populations (1, 2). Although the primary infection is almost always subclinically controlled, it may lead to the clinical syndrome of infectious mononucleosis (IM) (1, 3) when it occurs in adolescents and young adults.

IM is an EBV-driven proliferation of B lymphocytes that is controlled by humoral and cellular immune responses (1, 3). Characteristically there is a florid T cell response mainly consisting of activated CD8+ cytotoxic T cells specific for lytic, and to a lesser extent, latent viral antigens expressed on EBV-infected B cells (3, 4).

In an immunocompetent population, EBV remains latent for most of the host’s life. However, the viral load secreted in saliva can fluctuate over time and virions can be continuously released into saliva due to the viral reactivation process, which characterizes the switch from the latent to the lytic cycle of the virus (5). Additionally, a series of associations between terminal cell differentiation and EBV reactivation has been established (6), demonstrating that viral reactivation occurs mainly when the infected memory B cell is induced to differentiate into a plasma cell (7). Further, the transcription factors responsible for maintaining the memory B cell differentiation stage are described as repressors of lytic activation (8).

EBV is a ubiquitous herpesvirus and well-adapted to the human species, where most infections tend to converge to benign clinical outcomes. Although not being part of its natural replicative cycle, EBV is etiologically associated with the development of several neoplasms, highlighted by strong epidemiological and molecular evidences (9).

Solid and lymphoid neoplasms can be EBV-associated, such as nasopharyngeal carcinoma, gastric carcinoma, classical Hodgkin lymphoma, Burkitt lymphoma, post-transplant lymphoproliferative disease, extranodal NK/T-cell lymphoma (ENKTCL) and EBV-positive nodal T- and NK-cell lymphoma (NKTCL) (1, 10–12). It is estimated that approximately 200,000 new cases of EBV-associated tumours are diagnosed globally each year, which has led the International Agency for Research on Cancer (IARC) to consider it as group 1 carcinogen (9). The epidemiology of EBV-associated neoplasms is complex and may depend on age, sex, socioeconomic status, ethnographic customs, as well as host and viral genetic background (1, 6, 10, 13–15).

An established causal relation between EBV-infection of B cells and development of B cell lymphomas such as classical Hodgkin lymphoma and Burkitt lymphoma is well described (1, 12–14, 16, 17).

Much progress has been made in understanding how EBV transforms B cells and can contribute to their oncogenesis (1, 16–18). Meanwhile, since the first description of EBV association with the lethal midline granuloma (later renamed as ENKTCL), considerably less information has been published about its role in T and NK cell lymphomagenesis (19).

In lymphoid proliferations of T and NK cells, EBV is associated with ENKTCL, NKTCL, angioimmunoblastic- and follicular-type of nodal T-follicular helper cell lymphoma, systemic EBV-positive T-cell lymphoma of childhood as well as EBV-positive T- and NK-cell lymphoid proliferations (severe mosquito bite allergy, hydroa vacciniforme lymphoproliferative disorder and systemic chronic active EBV disease) (11, 18). Specially in the angioimmunoblastic- and follicular-type of nodal T-follicular helper cell lymphoma, EBV is detected mostly in the non-neoplastic B cells, while occasionally EBV-associated T cells, presumably reactive T cells, can be found in some cases (11, 18, 20, 21).

Due to a high incidence of ENKTCL and NKTCL in East Asian and Latin American populations a possible genetic predisposition has been suggested (18).

In this review we will discuss the genetic characteristics of EBV, its possible role in the oncogenesis of ENKTCL and NKTCL, two lymphomas of mature T and NK cells, as well as the main differences between these two lymphomas.

## The Epstein-Barr virus

EBV is a gammaherpesvirus and was the first human candidate oncovirus in 1964 (22). Subsequently it was identified as a ubiquitous herpesvirus worldwide and it took many years to establish its etiological role in several human cancers (9). Nowadays EBV is causally associated with lymphoproliferations of B- or T- cell origins and carcinomas, which could reflect the EBV cell tropism (23, 24).

### Viral latency

EBV exhibits complex survival and persistence dynamics, replicating its genome through the proliferation of infected B cells or production of the lytic virions. For this, the EBV expresses its genome differentially, characterizing the latency patterns (III, IIb, IIa, I and 0). The dynamics of EBV latencies were initially described in studies using B lymphoblastoid cell lineages and proposed as a germinal center model (25, 26). This model reflects mechanisms of viral adaptation through the host infection and it is characterized by successive downregulation of potentially immunogenic oncoproteins of EBV during the different stages of infection.

During the infection of immature B cells, the EBV genome, which is linear in the virion, circularizes through the fusion of its direct terminal repeats regions (TR), establishing itself in the episomal form (27, 28). In an initial moment, the virus begins to express a set of lytic genes in a transitory way, in a phase known as pre-latent abortive lytic cycle. In this phase there is no virion production or there is limited production of viral particles, which seems to occur due to the absence of methylation in the viral genome during this phase (29). Its known that the main lytic regulator of EBV, the Zta protein, preferentially binds to methylated DNA motifs, causing the transcription of lytic genes. During this initial phase that does not occur efficiently (30). Moreover this pre-latent abortive lytic phase proved to be essential for the establishment of latency in B cells (29).

Subsequently, the virus begins to express all of its latent genes. This phase of the infection is known as the growth program or the latency III profile and is characterized by the expression of six nuclear antigens (EBNA-1, -2, -3A, -3B, -3C and leader protein), three latent membrane protein (LMP-1, -2A and -2B), two untranslatable RNAs (EBER1 and EBER2, herein referred to as EBERs) and two clusters of microRNAs (BART and BHRF), which gather more than 40 sequences of miRNAs (26). The latency III pattern can be detected during the primary EBV infection in IM, lymphoblastoid cell lines and post-transplant lymphoproliferative diseases (31, 32).

The latency IIb may be observed during the transitional phase between latency III and IIa, and it is characterized by the expression of *EBNA1*, *EBNA2*, *EBNA3A*, *EBNA3C*, *EBERs*, BART miRNAs and BHRF miRNAs. This latency is observed in IM and post-transplant lymphoproliferative diseases too (31, 33, 34).

In the latency IIa expression of *EBNA1*, *LMPs*, EBERs and BART miRNAs takes place. This latency program is responsible for the survival of EBV-infected B cells in the germinal centre reaction, allowing them the possibility to differentiate into memory B cells. This latency can be found in classic Hodgkin lymphoma and nasopharyngeal carcinoma (31).

After differentiation into a memory B cell, the EBV-infected B cell in asymptomatic individuals exhibits the latency 0 which is characterized by the presence of only EBERs (31).

Latency I is detected during homeostatic proliferation of EBV-infected memory B cells, ensuring the viral persistence in the progeny. In this latency only *EBNA1*, EBERs and BART miRNAs are detected. Beyond EBV-infected memory B cells in cell division, the latency I is identified in Burkitt lymphoma (31). After cell division, the expression program will return to latency 0.

Although it varies between individuals, the frequency of infected cells in peripheral blood remains relatively stable, with a constant absolute number over several years in healthy carriers (35). Furthermore, despite this apparent stability in the number of infected B cells in the host, the viral load in saliva fluctuates over time, as a consequence of the viral reactivation process (36).

The mechanisms that lead to viral reactivation are not fully understood, but evidence suggests that it possibly occurs when the infected memory B cells are stimulated to differentiate in plasma cells, for example by stimuli such as cognate antigen recognition and interaction with T cells (37). Due to the transcription factors associated with plasma cell differentiation, such as XPB-1 (X-box binding protein 1) and BLIMP1 (B-lymphocyte-induced maturation protein 1), *BZLF1*, the main regulator of the switch from the latent cycle to the lytic cycle, is activated inducing the expression of other lytic genes and resulting in the formation of new viral particles (38).

The initial activation of the *BZLF1*, which encodes the lytic transactivator Zta, leads to the expression of another important gene at the beginning of the lytic phase, the *BRLF1* gene, which encodes the Rta transcription factor (39). Together, these two transcription factors drive the expression of a series of viral genes that enable and direct the amplification of viral DNA, as well as enzymes necessary for replication (40, 41). This process results in the production of new viral particles by EBV-infected plasma cells, which migrate mainly to the Waldeyer ring region. The virus is released and it is able to infect new immature B cells, restarting the replicative cycle and maintaining the B cell compartment of infected memory B cells. In addition the new viral particles can infect local epithelial cells, where the virus will replicate, and posteriorly be released in saliva with the potential to infect other hosts (42).

### Immune response

Taking into account the anti-EBV immune response, the viral proteins display a hierarchical immunodominance for the CD8+ T cell response. The strongest responses are induced by the proteins EBNA3A, EBNA3B, and EBNA3C, presenting in the latency III program (43, 44). A similar hierarchical response pattern is observed for the lytic cycle antigens, to which the strongest responses are observed against the immediate early antigens BZLF1, BRLF1 and BMRF1, also observed in the latency III (1, 43–45). These features allow the control of cells expressing the latency III program, consequently decreasing the deleterious potential offered by those proteins, which display high transforming power (43, 46).

The description of latency in the normal life cycle of EBV is only well characterized in B cells (32). Specially in IM, it is observed that different latency patterns can be found at the same time during the disease, possibly reflecting the dynamics of viral survival where the highly immunogenic latency pattern III is gradually replaced by the non-immunogenic latency pattern 0 (31, 47).

### The molecular characteristics of EBV

EBV is also known as human gammaherpesvirus 4 (or HHV-4), and like all human herpesviruses, is considered a biological agent with stable genetic material. However, the long period of coevolution with the host led to the development of viral adaptation control against the conditions imposed by the host’s antiviral defence (48, 49).

EBV possesses a complex double-strand DNA genome of approximately 172 kb, with the potential to translate more than 80 proteins and 46 functional small untranslated RNAs (EBERs, BART and BHRF1 miRNAs) (50). The majority of EBV’s transcripts are expressed only during the lytic cycle and eleven of them are transcribed during latency, of which only nine are translated. Moreover, EBV genome has several internal direct repeats, which are found in latency promoters and in short and long sequences throughout the genome, as well as TR at both ends of the genome (50). The presence of clonal TR from EBV in EBV-associated neoplasms suggests that the virus was present from an early stage, before the oncogenesis (50).

EBV harbours genes with high conservation among herpesviruses, such as genes encoding lytic cycle proteins involved in viral DNA replication, viral particle structure and viral DNA packaging. However, other genes are shared only among the *Gammaherpesvirinae* subfamily, for example those that encode immediate initial controllers of the lytic cycle, as *BZLF1* and *BRLF1*, and the latent proteins LMP1 and LMP2. Furthermore, some genes have similarities with the host, such as: *BZLF1*, *BHRF1* and *BCRF1* which are similar to the *c-FOS*, *BCL-2* and *IL-10* of the host, respectively (51, 52).

As the latency genes of EBV may be related with the development of some neoplasms, they have been used to characterize the viral diversity. The main objective behind this approach is to try to differentiate if restricted strains are truly associated with a neoplasia or if it reflects only a geographical restriction, prevailing in a specific population (53–60).

The EBV can be separated in two different genotypes (type 1 and type 2), based on the differences in the sequences of *EBNA-2* and *EBNA-3* (61). The main functional difference between these genotypes is that the type 1 is more efficient in establishing lymphoblastoid cell lineages *in vitro* when compared to type 2 (50). Despite the functional differences, it is observed that the EBV type 1 is highly prevalent worldwide when compared to type 2 (53, 62–64). The types are associated with geographic restriction due to immunocompetence of the population and/or group studied, rather than disease association (53, 62–64). Although the EBV types are not commonly associated with neoplasms *per se*, previous epidemiological studies associated the haplotype Type1+V3 with tumours in Southeast Asia and AIDS-associated lymphomas (65, 66). The V3 polymorphism is located in the promoter zone (Zp) of the lytic transactivator *BZLF1* gene, which is responsible for switch from latent to lytic cycle (65, 66). Recently it was demonstrated that this haplotype confers a functional increase in viral lytic reactivation, which could favour tumour development, since viral reactivation is a known risk for EBV-associated neoplasms (67, 68). Therefore, EBV type 1 together with other genetic viral factors may contribute to the development of EBV-associated malignancies.

Early efforts to describe EBV diversity led to a genetic characterization of various latent proteins (69). From these, LMP1 is a well-characterized latent protein with the ability to transform and immortalize not only B cells but also epithelial cells *in vitro* (64, 70). LMP1 protein has 386 amino acids, as well as three domains with different characteristics and functions, during the viral replicative cycle and cellular transformation. Moreover *LMP1* is one of the most variable EBV genes, displays a high intra-host variability, has a high genetic diversity and is geographically restricted, reflecting human migration over the past few centuries (54, 57, 60, 71, 72).

In the 1990s, the *LMP1* variant called CAO was characterized, harbouring specific polymorphisms such as 30 bp deletion (del30) located at the 3’end of the C-terminal domain, compared to the prototype (59, 73, 74). This CAO variant, in a model of overexpression, was able to induce neoplasia *in vivo* (73, 74). Since then, *LMP1* polymorphisms have been extensively studied in several groups of EBV-associated malignancies worldwide (54, 59, 60, 75, 76). Several polymorphisms and mutation hotspots, such as 15 bp insertion (ins15) that encodes a Janus Kinase 3 (JAK3) motif and the number of 33 bp repeats were associated to specific variants, as well as B cell lymphomas and AIDS-associated B cell lymphomas in specific populations (54, 59, 60, 75, 76).

### EBV-infection of T and NK cells

**Figure 1:**
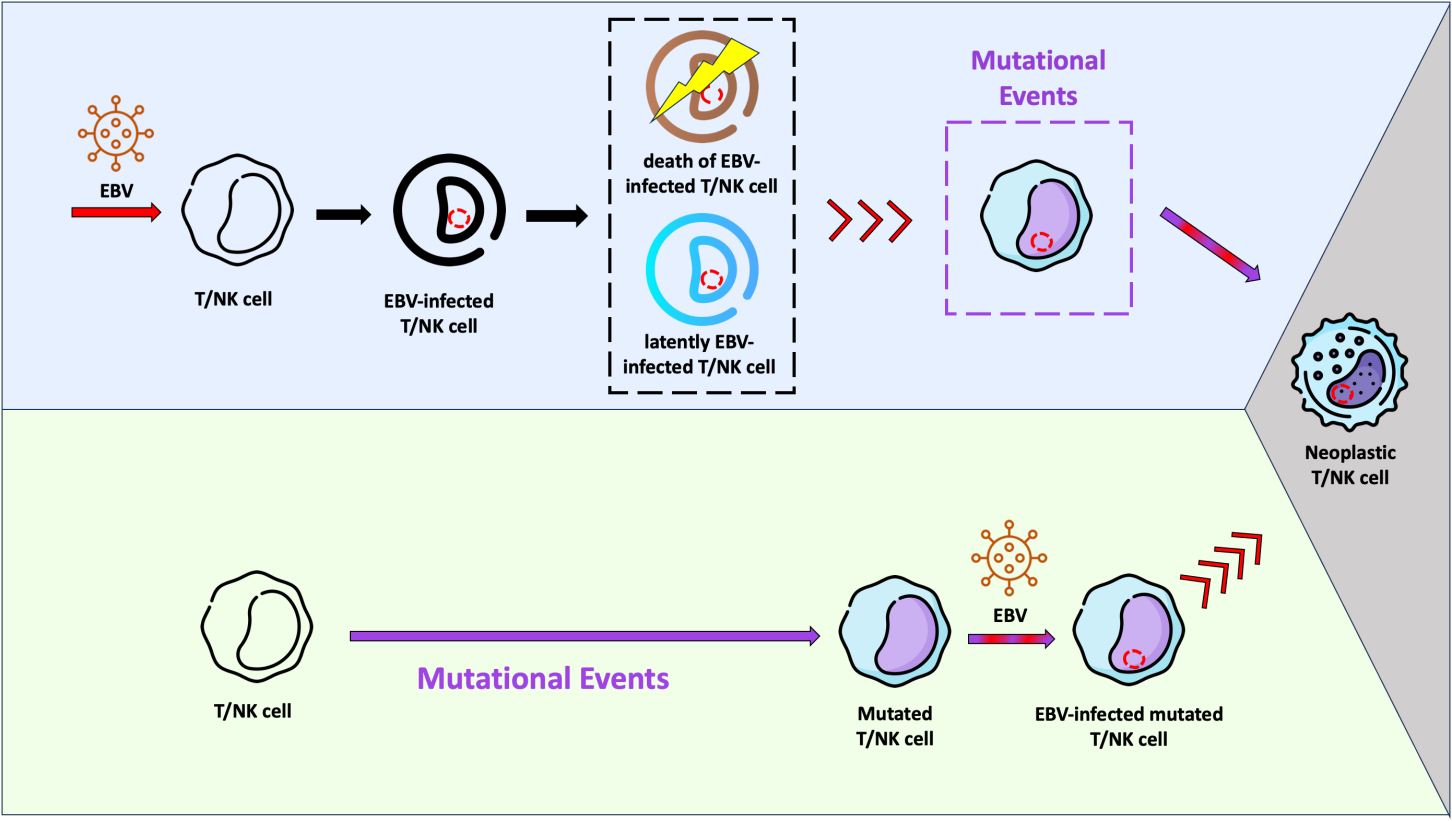
Schematic representation of two hypotheses related with the Epstein-Barr virus (EBV) infection and lymphomagenesis in T/NK cells. In the light purple background, a possible scenario is shown, where EBV infects “healthy” T/NK cells and stablishes a latent infection (cell in blue). It is possible that some of these infected cells are eliminated by the immune system or go into apoptosis (cell in brown). Viral reactivation phases with virus entry into the lytic cycle may occur in infected T/NK cells (red arrows), just as they happen in infected B cells. Mutational events in one EBV-infected T/NK cell may take place later, resulting in the fully malignant phenotype (grey background). In this context, EBV would act as an initiating agent. In the light green background, another hypothesis is presented. The EBV-infection occurs in a previously mutated T/NK cell (initiated cell) and the viral machinery would trigger the fully malignant phenotype (grey background). In this context, EBV would serve as a promoting agent.

We previously described that although B cells are the main compartment of EBV infection in IM, T and dendritic cells are also infected during the primary infection, however to a minor amount. In the same study we were able to show a predominance of EBV-infected CD8+ T cells over CD4+ T cells (47). Like T cells, NK cells can also be infected by EBV *in vivo* (77, 78). Moreover we showed that the EBV-infected T cells expressed EBNA1 and EBNA2 proteins, but not BZLF1, suggesting absence of lytic cycle (47). It is unknown whether these EBV-infected T/NK cells survive as a viral reservoir, go into apoptosis or are being destroyed by the immune system. The results described by Coleman et al, which EBV-infected T cells can be found in healthy Kenyan children at 12 months of age with persistence through 24 months of age, suggest that the EBV-infected T cells may survive (79). *In vitro* studies and studies using humanized mouse models have confirmed these observations (80, 81). Taking into account these studies, it is very likely that the infection of “healthy” T/NK cells by EBV *per se* is not sufficient to trigger the lymphomagenesis in these cells. Additional events may be necessary to the establishment of a fully malignant phenotype and consequently development of ENKTCL and NKTCL (**Figure 1**).

## ENKTCL

ENKTCL is an extranodal EBV-associated lymphoma of either NK or T cell lineage, which can affect nasal mucosa, skin, testis, kidney, gastrointestinal tract and salivary glands. As this lymphoma displays high prevalence in Asia and South America, it is hypothesized that population genetic characteristics, which influence the host immune response against EBV, may be related to lymphomagenesis (18, 82).

ENKTCL is characterized by a diffuse infiltrate of atypical lymphocytes of variable size, ranging from small to large. The nuclei are irregularly folded and hyperchromatic. In the small and medium atypical cells, the chromatin is granular, whereas in the large cells the nucleus tends to be vesicular. The nucleoli are usually small or inconspicuous. The amount of cytoplasm is moderate and frequently it appears pale to clear. Mitoses and apoptotic bodies are common. In most of the cases an angiocentric arrangement of tumour cells, together with angiodestruction and coagulative necrosis, is observed. Small lymphocytes, plasma cells, histiocytes and eosinophils are found in the background. Large areas of ulceration are noted in cases of mucosal presentation (11, 83).

Immunohistochemically the neoplastic cells are typically CD2+, CD56+, NKG2D+, NKG2A+, Tia1+, granzyme B+, perforin +, CD3-, CD4-, CD5-, CD8-, TCRαβ- and TCRγδ-, characterising the NK cell lineage (11, 84). CD7 shows a variable expression (11).

In the cases of true T cell lineage, the neoplastic cells are immunohistochemically CD2+, CD3+, CD5+, CD8+, Tia1+, granzyme B+ and perforin +. CD56 is normally negative, however can be positive. In addition there is the expression of TCRαβ or TCRγδ (11).

Cytogenetically ENKTCL are characterized by gains in 1p13, 2q33, 2q5, 3p14, 3q26, 6p21, 6p22, 7q34, 8q24, 9p24, 10q3, 13q4, 14q32, 17q21 and 22q11 as well as losses in chromosomes 1p4, 3q26, 5p13, 6q21-6q25, 8p22, 9p21, 12q3, 14q11, 14q21, 15q24, 17p13, 17p4, 18q22, 19q13 and 22q11 (85–88). Losses of 3q26 affected 50% of ENKTCL in one study, however abnormalities in this region are frequent in many neoplasms, including NKTCL and peripheral T cell lymphoma NOS (89, 90). Losses of 6q21-6q25 and the tumour suppressor genes present in that region, as *PODC3*, *PREP*, *PRDM1*, *ATG5*, *AIM1* and *HACE1*, are found in circa 20-41% of ENKTCL (86–89).

Regarding the mutational profile, the ENTKTCL displays frequent mutations in *JAK3*, *STAT3* and *STAT5b* (genes from JAK-STAT signalling pathway); in *EPHA1*, *GNAQ*, *NOTCH3*, *PTPRK* and *PTPRQ* (genes from RAS-MAPK signalling pathway); in *ARID1A, ASXL1*, *BCOR1, EP300, KMT2D* and *MLL2* (epigenetic modifiers); in *DDX3X* (a RNA helicase gene); in *MGA* and *TP53* (tumour suppressor genes); as well as in *FAS* (gene related to apoptosis) (85, 91–99).

Many overexpressed genes have been described in ENTKTCL and some of them are suspected to be involved in the pathogenesis of this lymphoma. *EZH2* may be overexpressed in the majority of ENKTCL. This gene has a dual function; it can act as a histone methyltransferase, inhibiting the protective role of tumour suppressor genes or it can activate genes involved in oncogenic pathways (100). In ENKTCL *EZH2* directly promotes cyclin D1 expression and this is related to MYC-mediated repression of miRNAs, such as miR26 and miR101, that normally target and inhibit *EZH2* expression (100). It is important to note that *MYC* is upregulated in ENKTCL and is a transcriptional target of EBNA1, EBNA2 and LMP1 (31). *RUNX3* is also overexpressed in ENKTCL, due to transcriptional action of MYC, inducing cell proliferation and reduced apoptosis (101). Other overexpressed genes are *AURKA* (occasioning cell proliferation) *PDGFRA* and *PD-L1* (contributing to immune scape) (87, 102, 103).

Additionally, promoter methylation may be a frequent event in ENKTCL with consequent silencing of tumour suppressor genes (such *ASNA*, *BIM*, *DAPK1*, *SOCS6*, *SHP1* and *TET2*) and regulators of cell cycle (such *CDKN1A*, *CDKN2A* and *CDKN2B*) (104, 105).

Recently Xiong et al. suggested that ENKTCL can be molecularly classified in three different molecular subtypes, named TSIM (from Tumour Suppressor and Immune Modulation), MB (from *MGA* mutation and LOH in the *BRDT* locus) and HEA (from mutations in *HDAC1*, *EP300* and *ARID1A*) (85).

The TSIM subtype is characterized by high expression of NK cell genes (*GZMB*, *KIR2DL1/2/4*, *KLRC1/2/3*, *KLRD1*, *KLRK1* and *NCR1/3*), mutations in genes of JAK-STAT pathway and in *TP53*, as well as amp9p24.1/*PD-L1/2* locus and del6q21. In this subgroup, an upregulation of *PD-L1/2* is found (85), which makes this group of patients feasible for checkpoint inhibitor treatment, at least from a theoretical point of view. Prospective studies are needed to confirm this hypothesis.

The MB subtype is distinguished by *MGA* mutation and 1p22.1/*BRDT* LOH. Both *MGA* and *BRDT* dysfunctions are associated with *MYC* amplification and clinically with tumour dissemination (85).

High expression of T cells genes (*CD3D/G*, *CD8A/B*, CD28, *ICOS* and VAV2/3), as well as mutation in *HDAC9*, *EP300* and *ARID1A* characterize the HEA subtype. Aberrant histone acetylation is the hallmark of this subgroup (85).

## NKTCL

NKTCL is a nodal EBV-associated lymphoma predominantly of T cells and more rarely of NK cells, which affects the lymph nodes (with or without extranodal involvement) and lacks nasal involvement (11). Clinically the patients are elderly or immunocompromised and display B symptoms as well as advanced stages (11). In the past, this lymphoma was known as EBV+ peripheral T cell lymphoma, not otherwise specified, and was part of the peripheral T cell lymphoma group. Now NKTCL is recognized as a distinct entity in the new WHO Classification (11).

Morphologically a diffuse and monomorphic proliferation of atypical cells of medium to large size with hyperchromatic nuclei and enlarged nucleoli, sometimes reminiscent of centroblasts is observed. Unlike ENKTCL, there is no coagulative necrosis and no angioinvasion (11).

Immunohistochemically the neoplastic cells are typically CD2+, CD3+ CD8+, Tia1+, granzyme B+, perforin + and CD56-, characterizing the T cell lineage. CD4 expression is unusual. The expression of TCRαβ or TCRγδ can be observed (11, 89, 106).

Cases of NK cell lineage are described and characterized by the expression of CD56, Tia1, granzyme B, perforin, as well as CD4-negativity. A small proportion of cases can co-express CD8 and CD56 (CD8+CD56+). In this situation, a clonality analysis should be performed to distinguish between T and NK cell origin (11, 89, 106).

Genetically, gains were found only in a small proportion of cases in the regions 1p13, 2q33, 3p14, 6p21, 6p22, 8q24, 14q32 and 22q11. Chromosomal loss seems to be a more recurrent lesion in this disease and it includes losses in 3q26, 6q24, 8p22, 9p21, 14q11, 17p13 and 22q11 (89, 106).

Regarding the mutational profile, NKTCL is characterized by frequent mutation in *TET2* followed by *PIK3CD, STAT3, DDX3X* and *PTPRD* (89). Mutation in *ATM, SETD2, JAK3, IRF4, STAT5B, DMXL2, MGA, FYN, LRP1B, FBXW7, FAT3, NOTCH3, LTK, CIC, FGFR2, MITF, KIT, SDHA, FANCD2, TNKS2, TOP2A, SLIT2, AXIN2, SYK, RAD54L, HSD3B1, MAPK2K4, GRIN2A, RBM10, FAT1* and *KDR* were also described by Wai et al, however in a very low frequency (89). Furthermore, mutations in *RHOA*, besides in *TET2*, were recently described in two cases of NKTCL with a T follicular helper cell phenotype (107).

Considering the gene expression profile, NKTCL is characterized by overexpression of many genes, including T-related genes (*CD2, CD8, CD3G, CD3D, TRAC, LEF1*), NF-κB-related genes (*BIRC3, NF−κB1, TLR8 and CD27*), *PD-L1*, *CD68* as well as downregulation of *CD56* (89, 106).

Moreover, the IL6/JAK/STAT3 signalling axis may be aberrantly hyperactivated in NKTCL, contributing to proliferation, survival, invasiveness and dissemination of neoplastic cells, as well as suppression of the antitumour immune response (89, 107, 108). The *PD-L1* upregulation in NKTCL may be associated to hyperactivation of IL6/JAK/STAT3 signalling, IFN−γ, as well as NF−κB pathway, and not related to amplification of 9p24 (89, 107). Considering these characteristics and the fact that IL6/JAK/STAT3 signalling axis is already therapeutically targetable, patients with NKTCL could benefit from targeted therapy (108).

The landscape of epigenetic alterations associated with NKTCL is still unknown (18, 89, 108, 109).

## A possible role of EBV in the oncogenesis of ENKTCL and NKTCL

Different EBV gene expression profiles reflect regulatory programs related to the lineage of infected host cells. Albeit EBV is detected in few T and NK cells in IM (47, 77, 78), the mechanism of infection in these cells type have not yet been completely elucidated. Some studies suggested that the infection of T and NK cells could occur through the immunological synapse, in an attempt by these cells to kill the EBV-infected B cells (23). We previously showed that EBV-infected T cells were mostly in contact with EBV-infected B cells in IM and the amount of EBV-infected T cells was directly related with the numbers of EBV-infected cells expressing PD-L1 (47). In addition, the molecule of HLA class II present on NK cells may interact with glycoproteins gp42 and gp85 from EBV, already described as fundamental in the internalization of EBV in HLA class II positive cells (110).

Recently it was demonstrated that CD21 cellular protein together with glycoprotein gp350 play an important role in the infection of the NK and/or mature T cells via trogocytosis, a mechanism that allows different cells to exchange pieces of their plasma membranes and suggested to occur in the interaction of mature T cells with malignant cells (23, 24).

Therefore, it is possible to think that the mechanism of EBV infection may be dependent on the cell type and the models so far established, using B cells, may not reflect what happens in other cell types. In consonance, EBV-infected T cells demonstrate *in vitro* a potential oncogenic distinct from those observed in LCLs, including different viral gene expression profile (111). Furthermore, previous studies *in vitro* demonstrated that T cells are possibly more permissive for the expression of immediate early viral genes than EBV-infected B cells (112, 113), with some evidence of this in EBV-associated neoplasia of T cell origin (114).

Although EBV has a dsDNA genome considered stable, genetic mechanisms that contribute to variability occur, such as point mutation, deletion, duplication, and intra/interstrain homologous recombination, as observed in *LMP1* (71, 72, 115). The del30 polymorphism of *LMP1* has been associated with diverse lymphomas worldwide (54, 61, 75). Moreover *LMP1* harbouring del30 may have a lower capacity to stimulate pro-inflammatory cytokine production, suggesting an immune escape ability, when compared to the prototype and del69 (116).

### EBV in ENKTCL

EBV is present in virtually almost all cases of ENKTCL and should be detected by EBER-ISH (11). Despite a relative consensus that this lymphoma seems to show latency I/II, a bone find characterization of latency pattern in this disease is still missing. The published studies have determined the viral latency with molecular-based methodology (as gene expression profile and RNA-Seq) (85, 89, 117, 118), which does not make it possible to assess which viral genes are being transcribed at the same time in the same cell.

A proportion of cases seems to exhibit the latency I, based on the identification of high levels of transcripts from EBNA1 only (85, 117). Another fraction of cases seems to show the latency II on account of the identification of high levels of transcripts from *EBNA1, LMP1, LMP2A, LMP2B, BNRF1, BILF1, BALF2, BALF3, BALF4, BALF5* and *BNLF2b* (85, 117). A minor number of cases seems to be in latency III due to the identification of high levels of transcripts from *EBNA1, LMP1, LMP2A* and *EBNA2* (89).

Interestingly, high level of antibodies against the proteins EBNA3A, BZLF1, BALF2, BMRF1, BVRF and BPLF1 (but not against EBNA1) have been detected in patients with ENKTCL (119), suggesting some degree of viral replication in these patients. The usefulness of these findings in the clinical management of ENKTCL is not yet established.

A precise *in situ* characterization of the viral latency in this disease, using multi colour immunohistochemistry or fluorescence *in situ* hybridization, as described for infectious mononucleosis (47), is still lacking. Furthermore, it remains to be determined whether all neoplastic cells show the same pattern of viral latency and which proportion of neoplastic cells could possibly show viral replication.

An apparent association of ENKTCL with EBV type 1 has been described (85, 120). However, EBV types *per se* are associated with specific populations than with diseases. EBV, specially type 1, is known to be highly frequent worldwide (121) and that association could represent an observational bias. Future studies focusing on the molecular characteristics of EBV and on host characteristics related to the anti-EBV immune response are needed to confirm that association. This notion is supported by some studies suggesting that few genetic regions of EBV may not be sufficient to understand the extent of EBV variation and its subsequent contribution to development of EBV-associated neoplasms (61, 122). The evaluation of different viral haplotypes needs to be included in further studies for a better understanding of EBV variants and their implications to the lymphomagenesis (61, 122).

Regarding the gene expression profile of EBV, this lymphoma subtype is enriched in the latent genes *LMP1/2A/2B*, *EBER1/2* and *EBNA1*, as well as lytic genes *BNRF1*, *BILF1*, *BALF5/4/3/2* and *BNLF2b* (85). Furthermore, single nucleotide variations in *BALF3* (G421R and T127A) may be prevalent in this lymphoma in relation to other EBV-associated diseases (85).

By considering the mutational profile of EBV, the del30 of *LMP1* is variable and depends on the population studied (120). Moreover small deletions in *EBNA2*, *EBNA3s* and *BLLF1/2* are common in ENKTCL (117). Frequent intragenic deletions affecting several BART micro-RNA clusters may be prevalent in ENKTCL (123). These deletions could impact the lytic cycle activation by eliciting the upregulation of *BZLF1* and *BRLF1*, which are downregulated by one of the BARTs miRNA.

Interestingly Xiong et al. found a correlation among EBV transcripts and their proposed molecular classification of ENKTCL (85). The TSIM subtype was associated with latency II pattern and high levels of *BALF3*; the MB subtype exhibited the lower levels of *LMP1* (as it is observed in the latency I pattern) and the HEA subtype correlated with latency II pattern and high levels of *BNRF1* (a protein necessary to latent infection) (85). In the same study the authors revealed that *BALF3* overexpression may cause DNA damage and contribute to genomic instability (85).

### EBV in NKTCL

EBV is also present in virtually all cases of NKTCL and should be detected by EBER-ISH (11). The EBV latency pattern in this lymphoma is not firmly established and only few studies have dealt with this, using only molecular techniques (as RT-PCR) (89, 114).

Apparently, the majority of cases displays latency II with expression of *EBNA1*, *BART*, *LMP1*, *LMP2A* and *LMP2B* (89, 114). A small proportion of cases exhibits latency III with additional expression of *EBNA2* (89, 114). One study demonstrated that the expression of the early lytic genes *BZLF1* was not accompanied by the expression of the late lytic genes *BHRF1* and *BLLF1* (114), suggesting an abortive lytic cycle.

Like in ENKTCL a precise *in situ* characterization of the viral latency in NKTCL, using multi colour immunohistochemistry or fluorescence *in situ* hybridization is also lacking. It is unknown in how far all neoplastic cells display the same viral latency pattern and which proportion of neoplastic cells may be in lytic cycle.

In a small cohort of patients from Hong Kong, it was demonstrated that all cases presented the del30 of *LMP1* and the majority of cases carried type 1 EBV (114). Results like this need to be interpreted with caution. As discussed previously, the EBV type 1 is more prevalent worldwide and currently it is impossible to stablish an unbiased association between this subtype and NKTCL. Moreover, it is unclear if the association between the del30 of *LMP1* and this lymphoma reflects a role of tumour cells in the origin and selection of this variant or if this is an observational bias, due to a possible higher prevalence of del30 of *LMP1* in healthy individuals from Asia. For example, in a small study including individuals from different regions from Thailand, a prevalence of del30 of *LMP1* in the Southern region was observed (124). Large studies including healthy individuals from Asia and/or other parts of the world, evaluating the prevalence of del30 of *LMP1*, are still lacking.

Compared to ENKTCL, NKTCL may exhibit lower expression of EBV miRNA (89). The exact meaning of this observation needs to be clarified.

Although NKTCL is strongly associated with EBV, both genomic and transcription profiles of the virus, as well as the characterization of the humoral immune response against EBV, have not been robustly explored in NKTCL.

### Viral proteins as candidates for target therapy

Although the therapeutic options for ENKTCL and NKTCL are not part of the objectives of this review in this research topic “Challenges in Peripheral T-Cell Lymphomas: from Biological Advances to Clinical Applicability” (125), we shall mention that epitopes derived of EBV proteins provide targets for immunotherapy in ENKTCL and NKTCL.

Adoptive immunotherapy with antigen-specific cytotoxic T cells (CTL) has been tested since the early 2000s for EBV-associated tumours and has been demonstrated to be safe (126–128).

As discussed previously, a hierarchical immunodominance for the CD8+ T cell response is observed against epitopes derived from proteins of the *EBNA3* family, *BZLF*, *BRLF1* and *BMRF1* (1, 43–45). Albeit less immunogenic, epitopes derived from *LMP1* and *LMP2* can be also used as potential targets for CD8+ T cells (128, 129). Considering the immune response of CD4+ T cells, the epitopes derived from *EBNA1* are the most immunodominant (43, 44, 128, 130, 131). The results published so far, regarding the characterization of latency pattern in ENKTCL and NKTCL, favour *LMP1*- and *LMP2*-derived epitopes as the best targets for adoptive immunotherapy with CTL.

Few studies using adoptive immunotherapy with CTL in ENKTCL have been published to date. All were phase 1 or 2; used *LMP1*- and/or *LMP2*-derived epitopes as target for the CTL (exception for the most recent, which included also *BARF1*- and *EBNA1*-derived epitopes); demonstrated no severe toxicity and exhibited objective responses in most of the cases, characterized by disease stability or remission during the follow-up time of the studies (132–135). These results are encouraging and point to the necessity of optimising this therapeutic option.

Currently there are no published studies on the use of CTL in NKTCL.

### EBV viral load

The detection and quantification of circulating EBV DNA have been used in the diagnosis and management of EBV-associated neoplasms (136). Plasma and whole blood can be used to this quantification, with a good correlation between them. However, the optimal source of viral DNA remains uncertain, due to the limited number of studies comparing the two methodologies (136). *EBNA1*, *BamH*I and *LMP2* are commonly used as target for the viral load evaluation in real-time PCR-based assays (137–141).

Specially in ENKTCL, assessing the viral load in plasma seems to be more useful, since this methodology appears to reflect the tumour burden (137–139, 142). Moreover, high levels of EBV DNA load have shown a close correlation with a worse clinical outcome and prediction of early relapse (137–139).

The impact of EBV viral load on the clinical management of NKTCL is so far unknown.

## Human leukocyte antigens in ENKTCL and NKTCL

Besides the EBV *per se*, the genetic background of the host, related to the anti-EBV and/or anti-tumour immune response, may also have an influence on the development of ENKTCL and NKTCL. It is well established that the anti-viral immune response is dependent on major histocompatibility complex (MHC) presentation of viral antigens (143–145).

During the primary EBV infection in patients with IM, the immune response is mainly characterized by a large expansion of EBV-specific CD8+ T cells. Response against immediate early and early lytic EBV epitopes may constitute half of the CD8+ T cells population (146). A directed immune response to late proteins is less frequent and in small amount, which directly impacts any future viral reactivation (146). Different levels of immunodominance may reflect the time which different epitopes are presented on the surface of infected cells. As the lytic cycle progresses the cell’s antigen-processing capacity may be increasingly impaired by the set of viral immune evasion proteins (146). In this way, it is suggested that the main immune response against EBV is driven by direct CD8+ T cell contact with lytically infected cells, and this interaction is depending on MHC characteristics and EBV epitopes (44, 46).

In humans, the MHC is known as human leukocyte antigen (HLA), and it exhibits high polymorphism. Many studies have shown that different HLA haplotypes are associated with different types of diseases, such as autoimmune diseases and neoplasms (145, 147–153).

Regarding ENKTCL, the haplotype 47F-67I from *HLA-DRB1* may be associated with reduced risk of lymphoma development, while the haplotype 47Y-67L may be associated with increased risk in patients from various countries of East Asia. In the same study, the authors suggested that *HLA-DPB1* and *HLA-DRB1* are the two major genes independently conferring individual risk to ENKTCL (82). The β chain, which forms HLA-DR heterodimers with α chain, is encoded by *HLA-DRB1* (147). The different antigen binding affinities of the HLA-DR complex impact its ability to present extracellular antigens to CD4 T cell lymphocytes, influencing the immune response against EBV and/or tumour antigens (82, 147).

The association between variants of *HLA-DRB1* and ENKTCL must be confirmed by other robust studies. Furthermore, specific studies comparing the HLA subtypes between cases of NKTCL and controls are still lacking.

## Conclusions

Although molecular, clinical and immunohistological differences are well established between ENKTCL and NKTCL, allowing the diagnostic difference between these neoplasms in clinical practice, the etiopathogenic role of EBV in these lymphomas has not yet been elucidated. In the light of current knowledge, there are more questions than answers.

The ability of EBV to infect T/NK cells during the primary infection is well characterised, as discussed before. Considering the studies published so far, it is very likely that this infection in T/NK cells *per se* does not trigger the lymphomagenesis *in vivo*, in the same way that it does not for EBV-infected B cells either. In other words, the EBV would act as an initiating agent, which can transform the cells it infects, however subsequent additional cellular events may be required for the fully malignant phenotype (**Figure 1**) (1, 154). Nonetheless, it is not possible to exclude a promoting role of EBV in the lymphomagenesis, as a consequence of its infection in previously mutated T/NK cells (**Figure 1**). The scenario in which ENKTCL and NKTCL can develop is still unknown.

May EBV-infected T/NK cells with persistent latency pattern II or III be the most susceptible to the oncogenic events? For example, ENKTCL is enriched in transcripts of latent EBV genes such as *LMP1/2A/2B*, as well as transcripts of lytic genes, including *BNRF1*, *BILF1*, *BALF5/4/3/2* and *BNLF2b* (85), which possess the ability to interfere with the host cell machinery. May recombination and/or mutation events of the EBV genome in some T/NK cell be responsible for the oncogenesis (or at least the initial event)? Specific T cell epitope mutations of EBV, favouring the immune evasion, have been described in ENKTCL (117). Furthermore, it is still unknown whether the molecular profile of EBV in neoplastic cells of ENKTCL and NKTCL is the same or not as that present in non-neoplastic EBV-infected cells of the same host.

No less important are the genetic characteristics of the host, specially those related to the anti-EBV immune response, which still need better characterisation in ENKTCL and NKTCL. Is it possible that the immune inability to recognise and destroy any EBV-infected T/NK cells in persistent latency pattern other than 0 is a major factor in the lymphomagenesis? Some grade of immunodeficiency (as immunesenescence), for example, is present at diagnosis of NKTCL (11).

Nearly sixty years after the discovery of EBV, this virus still remains intriguing.

## Author contributions

MHMB: conceptualization, writing, review and editing. PDSA: writing and review. All authors contributed to the article and approved the submitted version.
